# How Family's Support of Perseverance in Creative Efforts Influences the Originality of Children's Drawing During the Period of COVID-19 Pandemic?

**DOI:** 10.3389/fpsyg.2021.600810

**Published:** 2021-02-09

**Authors:** Bowen Shi, Ziwei Xing, Mei Yang, Chaoying Tang

**Affiliations:** ^1^CAS Key Laboratory of Behavioral Science, Institute of Psychology, Chinese Academy of Sciences, Beijing, China; ^2^Department of Psychology, University of Chinese Academy of Sciences, Beijing, China; ^3^School of Economics and Management, University of Chinese Academy of Sciences, Beijing, China

**Keywords:** originality, search persistence, pandemic exposure, prosocial motivation, family's support of perseverance in creative efforts

## Abstract

This study points out that families' support of perseverance in creative efforts will increase children's originality of creative drawing through children's persistence in information searching. Data analysis based on 134 Chinese young children's creative drawings and survey supports the above hypothesis. Moreover, children's exposure to COVID-19 pandemic positively moderates the relationship between supporting perseverance and children's search persistence, such that high exposure to COVID-19 pandemic will increase the positive relationship between support of perseverance and search persistence. And children's prosocial motivation inhibits the influence of search persistence on originality. Contributions to the theory of children's creativity are discussed.

## Introduction

“Creativity,” defined as the originality and usefulness of outputs (Plucker et al., 2004), is important for children's growth and success in the future. Children's creativity is a well-established research topic (Jackson et al., 2012; Krumm et al., 2018). In the creativity research literature, the creativity of children is usually represented by their divergent thinking and problem-solving skills (Runco and Acar, 2012), which have been assessed based on their works of art, creative drawings, and creative ideas. A frequently-used assessment method of creative products is the Consensual Assessment Technique (CAT) (Amabile, 1982; Baer et al., 2004). Among them, originality—the ability to generate unusual or unique outputs—is considered as a key aspect of creativity (Grace and Maher, 2014) or even the most important predictor of creativity (Rothenberg and Hausman, 1976; Runco, 1988; Runco et al., 2005; Acar et al., 2017). However, little attention has been given to originality and its potential predictors, especially among children.

**Figure 1:**
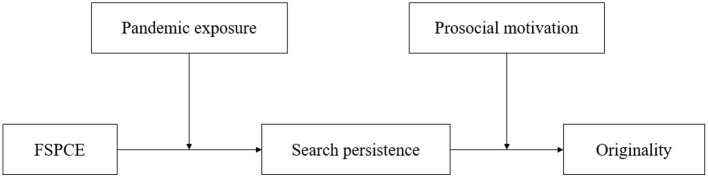
Theoretical model of the study. FSPCE = family's support of perseverance in creative efforts.

Meanwhile, the COVID-19 pandemic has created an extraordinary period during which parents and children are being requested to stay at home in order to curb the spread of the virus. According to attachment theory (Bowlby, 1958, 1988), family members may interact with each other more frequently and parents have a greater influence on children when asked to stay at home. In this period, children's behaviors and the originality they manifest may be affected by the family environment and social environment (exposure extent to the pandemic). However, little research was conducted to investigate the relationship between family influence, student behavior and originality and whether this relationship could be affected by the pandemic and personal trait. To address this gap, our study focused on family's support of perseverance in creative efforts (FSPCE), one dimension of family creativity climate, and its effect on children's search persistence and originality. We propose that FSPCE is positively associated with search persistence, and children who have high search persistence create high original outcomes. We argue that under the context of high FSPCE, children would be affected by parents' encouragement of creative efforts and put more time and effort into searching for information to make great works. With more information children obtain, they increase relevant knowledge and insights which help them more likely to make original works. We also suppose pandemic exposure would enhance this relationship. Attachment theory argues when families are experiencing unusual stress, children tend to activate their attachment system (Newman and Newman, 2020). In this case, the epidemic may make children more close to parents and increase the family influence. We also propose children's prosocial motivation may suppress the relationship between search persistence and original outcomes as paying attention to others' benefits make children less deviate from routine which may restrict their works' originality (Beersma and De Dreu, 2005). In this study, we collected 134 Chinese young children's creative drawings and surveys to empirically test the model (see in Figure 1). The study also makes contributions to the attachment theory and original studies.

### Originality, Search Persistence, and Family's Support of Perseverance in Creative Efforts

Our study focuses on “search persistence,” defined as the extent to which people continue to gather information (Li et al., 2013), as a predictor of originality. The main reason is that persistent searchers are able to obtain knowledge (Amabile, 1983; Nonaka and Takeuchi, 1995; Hass and Burke, 2016), which comprise the core element of new ideas (Hambrick and Mason, 1984). Persistent searchers have better skills in categorizing information, understanding information, and finding more connections among different ideas (Li et al., 2013). The literature has shown that idea generation processes are the result of diversified information and knowledge (Nijstad et al., 2002). Searching new knowledge and information is a critical factor for the generation of new ideas (King and Markant, 2020). In fact, authors who have integrated diverse experiences and knowledge generated highly original books (Taylor and Greve, 2006). Exploring and integrating diverse knowledge also broaden the range of novel solutions for a given problem (Amabile, 1983, 1997) along with the potential to make an uncommon solution (Fredrickson and Branigan, 2005).

This is especially the case for young children, whose knowledge base is weak so they need to acquire new knowledge to finish creative tasks. A similar study found that exposed information promoted children's divergent thinking in TTCT activities, which implicitly include originality (Clapham, 2001). The creative task of the current study is a creative drawing task that is closely related to the real world; thus, children can improve their understanding toward the topic by collecting related information and knowledge. Hence, we present the following hypothesis:

**Hypothesis 1**: Search persistence positively influences the originality of children's creative drawings.

Family's support of perseverance in creative efforts is a dimension of creative family climate, which means parents engage in activities that strengthen children's perseverance in the performance of creative tasks, such as providing verbal and emotional support, tolerating failure, and helping children cope with difficult circumstances (Kwaśniewska et al., 2018). FSPCE has been found to be positively associated with student creativity (Kwaśniewska et al., 2018). In the current study, we propose that FSPCE will influence originality through children's search persistence. There are several reasons for this assumption. First, creativity requires perseverance and effort (Rojas, 2015). Extant studies have shown that parents who support their children's perseverance in creative endeavors help improve their children's learning (Kotaman, 2018). In addition, children's beliefs and motivations (Alberto Valdes-Cuervo et al., 2020) as well as their academic performance are influenced by the support of their parents (Amabile, 1996; Gonida and Cortina, 2014; Silinskas and Kikas, 2019). Support from parents also motivate children to work harder (Alberto Valdes-Cuervo et al., 2020), thus promoting greater effort and more courage in conducting long-time learning (Dornyei and Ryan, 2015). Therefore, we argue that FSPCE will encourage children to constantly search for information and knowledge during the process of creative work. Thus, the following hypothesis is presented:

**Hypothesis 2**: FSPCE is positively associated with search persistence.

### The Moderating Effects of Pandemic Exposure and Prosocial Motivation

We also assume that the influence of family climate on originality will be strengthened if children's exposure to COVID-19 pandemic is high. On the one hand, according to attachment theory (Bowlby, 1958, 1988), when families are experiencing unusual stress, children of any age tend to activate their attachment system (Newman and Newman, 2020). That is, the experience of stressful events can lead to more attachment behaviors in children. After a disaster, young children are expected to increase their sense of closeness to the caregiver (Lieberman and Amaya-Jackson, 2005) and their need to be in proximity with their family (Van Bavel et al., 2020). As such, during a disaster, parents will have more influence on their children (Osofsky, 2002). The COVID-19 pandemic is a massive global public health crisis, which we suggest, could lead children to be more closely attached to their families. As such, the influence of family environment on children's behavior will be strengthened. During this period, support from parents will also help children reduce the intense emotions associated with the event and encourage them to put more focus on learning (McCubbin and Patterson, 1985; McKenry and Price, 2000). A similar study on college students under job search pressure found that parental support significantly influenced students' job search behaviors (Jang and Yoo, 2014).

On the other hand, uncertainty reduction theory points out that high levels of uncertainty increases information-seeking behavior. As uncertainty levels decline, information-seeking behavior also decreases (Berger and Calabrese, 1974). Seeking information is one of the methods children and adolescents use to deal with stress (Compas et al., 2001) and reduce uncertainty (Atkin, 1973; Baum et al., 1981). As information is needed for predicting and improving one's ability to deal with current and future challenges (Baum et al., 1981), gaining more information can increase one's sense of “being in control” (Janis, 1968). In addition, emotional responses, such as worry and anxiety, are the strongest predictors for seeking additional information (Turner et al., 2006; Ter Huurne and Griffin, 2007). The uncertainty caused by external threats is usually accompanied by feelings of anxiety, which drive people to seek more information (Kelly et al., 1963; Afifi and Weiner, 2006). In a study on external safety risks, Griffin et al. and his coworkers found that the anger caused by a flood-related disaster encouraged more information search behaviors among the study participants (Griffin et al., 2008). Taken together, we thus present the following hypothesis:

**Hypothesis 3**: Pandemic exposure will moderate the relationship between FSPCE and children's search persistence, such that when children's pandemic exposure is high, the positive relationship between FSPCE and search persistence will also be high.

We argue that children's prosocial motivation will negatively moderate the correlation between search persistence and originality. “Prosocial motivation” refers to one's desire to expend effort to benefit other people rather than oneself. Prosocial people are more likely to think from other people's perspectives (Eisenberg and Miller, 1987) and can easily develop useful solutions (Grant and Berry, 2011). Moreover, prosocial people value harmony and prefer to reduce uncertainty by either adopting accustomed practices and frameworks (Mueller et al., 2012) or avoiding engaging in contentious behaviors (Dreu et al., 2000). Thus, they tend to do better work on convergent thinking rather than divergent thinking (Beersma and De Dreu, 2005). A similar study on negotiation found that group members with a prosocial motivation generated more useful ideas than members with a pro-self motivation (Beersma and De Dreu, 2005).

In addition, the pursuit of a high level of originality requires a shift to more individualistic tendencies (Munoz et al., 2020) and deviation from routines and the status quo (Zhou and George, 2001; Anderson et al., 2014). However, individuals with prosocial motivations are more likely to think from the perspectives of others and deviate less from the routine (Beersma and De Dreu, 2005), which may limit their divergent thinking and lead to cognition closure (Miron-Spektor and Beenen, 2015). Cognitive closure enables individuals to focus on a feasible solution, prohibits them from exploring alternative solutions (Chirumbolo et al., 2005), and restricts the ideational fluidity early in the creative process (Guilford, 1950). Thus, individuals with high level of cognitive closure produce fewer and less original ideas than those with low level of closure (Chirumbolo et al., 2005). As mentioned above, search persistence can bring about original solutions mainly due to the fact that it is accompanied by the act of accessing new knowledge and gaining other perspectives (Hirst et al., 2009). Thus, the need for usefulness, accompanied by cognitive closure and prosocial motivation, will prevent children from taking full value from their accessed information and knowledge. Therefore, we expect that prosocial motivation will moderate search persistence and originality. In relation to this, we present the following hypothesis:

**Hypothesis 4**: Children's prosocial motivation will moderate the relationship between search persistence and the originality of their creative drawing, such that when prosocial motivation is high, the positive relationship between search persistence and originality will be low.

## Methods

### Procedures and Participants

In February 2020, the Chinese Creative Teaching Association (CCTA) organized a creative drawing competition of with the topic of “Protecting Humans, Fighting COVID-19.” The competition required the participants to draw a creative figure related to the topic and provide a short description of <200 words to explain their drawing. Children were also required to sign a form expressing their agreement to the disclosure of their creative drawings publicly. More than 2,000 Chinese children submitted their creative drawings online. For some sample drawings, see Appendix. We obtained the support and permission of the CCTA to randomly select the participating children and invite them to accomplish an online survey using a mobile phone app developed by the CCTA. In this way, children can answer the questionnaire by simply choosing the answers on their mobile phones. The children read the informed consent form first before they were asked to answer the questionnaire.

The questionnaire was divided into two parts: one was for children, and the other was for their parents (they were not included in this study). All the children, who joined voluntarily, were told that the survey activity was for an academic study on family and creativity and that all individual data would be kept confidential. They were asked to answer the questions about family climate, pandemic exposure, and search persistence. They were then asked to input the ID number they used in the creative drawing competition. The online survey system was kept open for 2 weeks. Within the 2-week survey, 134 children joined our study and answered the questionnaire. Among all children surveyed, 40.3% were male and 59.7% were female. In terms of grade level, 89.6% were elementary school children, 8.2% were junior middle school children, and 2.2% were pre-primary children. About 69.4% of the children were the only child in their respective families, and 29.1% came from families with two children.

All of the procedures performed in the study involving human participants were approved by the Chinese Creative Teaching Association. This study observed the voluntary, confidentiality, safety, and compensation principles as well as other internationally accepted principles of ethical review.

### Measurement

#### Originality

We used CAT to assess the originality (Amabile, 1982). The CAT is frequently used for evaluating creative products (Kaufman et al., 2008). In our study, raters were three graduates majored in creativity (one male and two female). They were just finished a course of creativity which included the introduction of CAT and chances to assess creative products by using CAT. Before they rated the drawings of this study, they got 1-h training by a professor in creativity field on how to assess originality and usefulness by CAT. Then they rated the sample four drawings independently, and the professor set up an online meeting and gave them another 1-h instruction of how to rate the originality and usefulness in order to make sure that they got necessary expertise. Then, three graduates got the new drawings of this study and rated them independently. Originality was rated by the 5-point Likert scale, ranging from 1 (very low original) to 5 (very high original). The intraclass correlation among evaluators was 0.84, which meant their scores have good consistency. The data of originality in this study are the average scores of the three raters.

#### Family's Support of Perseverance in Creative Efforts (FSPCE)

Two items of FSPCE came from Kwaśniewska et al. (2018): “My parents show me that making mistakes is natural” and “My parents attentively accompany me through failures and let me realize that failures give me valuable lessons.” The Cronbach's alpha coefficient in this study was 0.64.

#### Search Persistence

To assess search persistence, a three-item scale was used, which was adapted from the search persistence scale developed by Li et al. (2013). A sample item is “During the process of completing the creative drawing, I take as much time as needed to identify all available information.” The Cronbach's alpha in this study was 0.84.

#### Pandemic Exposure

This index consisted of three items adapted from the Disaster Exposure Scale to assess pandemic exposure (Drury et al., 2016). A sample item is “When working on the creative painting, I was directly affected by the pandemic.” Cronbach's alpha in this study was 0.90.

#### Prosocial Motivation

We assessed prosocial motivation with a three-item scale adapted from previous literature (Grant and Sumanth, 2009). An example item is “I like to work on tasks that have the potential to benefit others.” Cronbach's alpha in this study was 0.78.

All items of FSPCE, search persistence, pandemic exposure, and prosocial motivation, were rated using a 5-point Likert scale, ranging from 1 (strongly disagree) to 5 (strongly agree). Back-translation method was used.

#### Control Variables

We controlled five demographic variables: gender, grade level, parents' highest education, parents' age, and the number of children in the family. Previous literature has shown an inconsistent effect of gender on creativity (Martín-Brufau and Corbalán, 2016). Age has also been identified as a key factor in the development of creativity among children (Yeh and Li, 2008). As parents and siblings have various influences on children's creativity (Gulliksen, 2018; Pang et al., 2020), parents' educational level, parents' age, and the number of children in the family were also controlled.

The usefulness of the drawings was also controlled in this study. Usefulness was also assessed by using CAT. The intra-class correlation was 0.73, which meant good consistency among raters. Studies have found the overlap of neural basis and brain activities between appropriateness and originality (Huang et al., 2015, 2018). Moreover, for creative ideas, the high score of usefulness does not necessarily mean low score of originality. However, usefulness is often rated based on perception of familiarity, which might come at the expense of originality (Berg, 2014). Thus, the usefulness score might be associated with the originality score. In order to exclude this potential interference, usefulness was thus controlled in this study.

### Analysis Strategy

We first calculated the means, standard deviations, and correlation matrix. Then, we used linear regression to test the hypotheses and the PROCESS macro (model 21), which was developed by Hayes (2012), to test the moderated mediation model. Additionally, for examining the indirect and conditional indirect effects, we drew on the bootstrapping method (Hayes and Scharkow, 2013) to create a 95% bias-corrected confidence interval (CI) from 5,000 resamples. The effects were significant when the confidence interval did not include zero.

The measures of four variables (FSPCE, pandemic exposure, search persistence, and prosocial motivation) in this study are self-reported, which potential arises common method bias. So, we used Harman's single factor test to examine the bias. It turns out that the first (biggest) factor explained 36.28% of variance, which is lower than the cutting-off point of 50% and indicates that the common method bias in this study is not a big concern (Podsakoff and Organ, 1986).

## Results

The theoretical model of the study is shown in Figure 1. The means, standard deviations, and correlations are reported in Table 1. Next, we performed confirmatory analysis on the questionnaire items. We compared the fitness index of the four-factor model (FSPCE, pandemic exposure, search persistence, and prosocial motivation), the three-factor model (FSPCE and search persistence as one factor), the two-factor model (SPCE, search persistence, and prosocial motivation as one factor), and the single-factor model (see Table 2). Results showed that the four-factor model indicated significantly better fit than the other models, thus supporting the proposition that all measures can be differentiated.

**Table 1:** Means, standard deviations, and correlations.

**Variable**	***M***	***SD***	**1**	**2**	**3**	**4**	**5**	**6**	**7**	**8**	**9**	**10**	**11**
1.Gender	0.40	0.49	1										
2.Grade	3.13	10.97	−0.11	1									
3.Parents' highest education	2.56	0.95	0.01	−0.17	1								
4.Number of children	1.35	0.65	0.02	−0.04	−0.23^**^	1							
5.The closest parent's age	38.54	40.20	0.10	0.31^**^	0.03	0.17	1						
6.FSPCE	4.28	0.58	0.12	0.07	0.13	−0.01	0.07	(0.64)					
7.Pandemic exposure	3.40	10.08	0.07	0.09	0.03	−0.07	−0.02	0.25^**^	(0.84)				
8.Search persistence	3.97	0.71	0.11	0.00	0.02	0.02	−0.06	0.30^**^	0.44^**^	(0.90)			
9.Prosocial motivation	4.25	0.56	−0.03	0.06	0.13	−0.08	−0.01	0.40^**^	0.18^*^	0.34^**^	(0.78)		
10.Originality	2.58	1.08	−0.00	−0.02	0.11	−0.07	0.02	−0.07	0.06	0.12	−0.06	(0.84)	
11.Usefulness	2.19	0.97	−0.02	0.07	0.03	0.03	−0.04	0.05	−0.112	−0.12	−0.06	−0.45^**^	(0.73)

N = 134

** *p < 0.01 (2-tailed)*.

* *p < 0.05 (2-tailed). Numbers on the diagonal represents Cronbach's alpha of each variable. To originality and usefulness, numbers on the diagonal represents intra-class correlation. FSPCE, family's support perseverance in creative efforts*.

**Table 2:** Confirmatory analysis results of all measurements in the study.

**Model**	**χ2**	***df***	**Δχ2**	**Δ*df***	**CFI**	**SRMR**	**RMSEA**	**TFI**
Four-factor model	57.47^*^	38	—	—	0.97	0.06	0.06	0.96
Three-factor model	109.21^**^	41	51.74^**^	3	0.90	0.10	0.11	0.87
Two-factor model	194.09^**^	43	136.62^**^	5	0.78	0.12	0.16	0.72
One-factor model	371.71^**^	44	314.24^**^	6	0.53	0.19	0.24	0.42

** *p < 0.01 (2-tailed)*.

* *p < 0.05 (2-tailed). CFI, comparative fit index; SRMR, standardized root mean square residual; RMSEA, root mean square error of approximation; TLI, Tucker-Lewis index*.

*Four-factor model: full model*.

*Three-factor model: combing FSPCE and search persistence as one factor*.

*Two-factor model: combing FSPCE, search persistence, prosocial motivation as one factor*.

*One-factor model: combing all measures as one factor. FSPCE, family's support of perseverance in creative efforts*.

The regression model's results are shown in Table 3. As can be seen, the FSPCE was positively related to search persistence (β = 0.50, *t* = 4.61, *p* < 0.01). However, there was no significant relationship between search persistence and originality after controlling the covariates (β = 0.20, *t* = 1.37, *n.s*.). Thus, **Hypothesis 1 was not supported, but Hypothesis 2 was supported**. Further, pandemic exposure moderated the relationship between FSPCE and search persistence (β = 0.09, *t* = 2.09, *p* < 0.05), thus **supporting Hypothesis 3**. Model 5 in Table 2 shows the marginally significant interaction between search persistence and prosocial motivation (β = −0.20, *t* = −1.99, *p* < 0.05), thus **supporting Hypothesis 4**.

**Table 3:** Regression results for the hypothesis model.

	**Search persistence**			**Originality**			
**Predictors**	**Model 1**	**Model 2**	**Model 3**	**Model 1**	**Model 2**	**Model 3**	**Model 4**
Gender	0.15	0.07	0.06	−0.02	−0.02	−0.05	−0.05
Grade	0.04	0.01	0.00	0.02	0.02	0.02	0.04
Parents' highest education	0.07	0.03	0.03	0.15	0.15	0.16	0.15
number of children	0.06	0.09	0.08	−0.05	−0.05	−0.07	−0.03
The closes parent's age	−0.02	−0.01	−0.01	0.00	0.00	0.00	−0.01
Usefulness	−0.10	−0.08	−0.06	−0.51^**^	−0.51^**^	−0.50^**^	−0.51^**^
FSPCE		0.46^**^	0.50^**^		−0.05	−0.01	0.02
Pandemic exposure		0.21^**^	0.19^**^				
FSPCE × Pandemic exposure			0.09^*^				
Search persistence						0.18	0.20
Prosocial motivation						−0.28	−0.24
Search persistence × Prosocial motivation							−0.20^*^
*R^2^*	0.05	0.31	0.33	0.22	0.22	0.24	0.27
Δ*R^2^*		0.26^**^	0.02^*^		0.00	0.02	0.02^*^

N = 134

** *p < 0.01 (2-tailed)*.

* *p < 0.05 (2-tailed). FSPCE= family's support of perseverance in creative efforts*.

**Figure 2:**
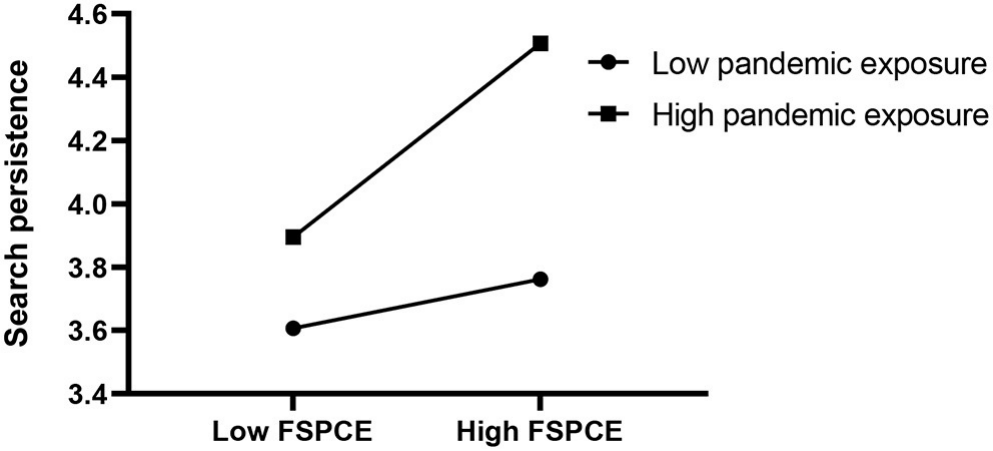
The moderating effect of pandemic exposure on FSPCE (family's support of perseverance in creative efforts) and search persistence.

**Figure 3:**
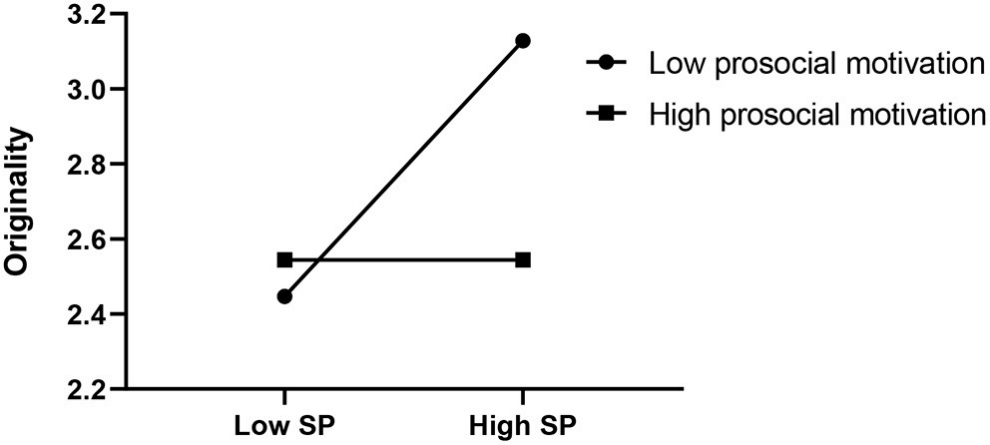
The moderating effect of prosocial motivation on SP (search persistence) and originality.

Next, we plotted the simple slopes, which predicted the relationship between FSPCE and search persistence, as moderated by pandemic exposure, as well as that between search persistence and originality, as moderated by prosocial motivation. As presented in Figure 2, the slope of the association between FSPCE and search persistence was relatively strong for participants with high pandemic exposure (β = 0.65, SE = 0.16, *p* < 0.01), whereas the slope was not significant with low pandemic exposure (β = 0.25, SE = 0.13, *n.s*.). Additionally, as shown in Figure 3, the effect of search persistence on originality was enhanced in children with low prosocial motivation (β = 0.48, SE = 0.21, *p* < 0.05) compared to children with high prosocial motivation (β = −0.08, SE = 0.19, *n.s*.).

Next, in order to test moderated mediation effect, we used PROCESS macro with 5,000 resamples. Conditional indirect effects are shown with two moderators combined at average value (Mean), high level (+1 SD), and low level (−1 SD), following Hayes (2015) method for testing moderated mediation. As shown in Table 4, when pandemic exposure was low, the indirect effects were not significant regardless of the extent of prosocial motivation. For children with average pandemic exposure, the indirect effects were significant with low prosocial motivation (Effect = 0.24, SE = 0.12, 95% CI = [0.02, 0.50]). For children with high pandemic exposure, the indirect effects were relatively stronger with low prosocial motivation (Effect = 0.33, SE = 0.17, 95% CI = [0.03, 0.66]) compared to average and high prosocial motivation.

**Table 4:** Summary of indirect effects of FSPCE on originality via search persistence.

	**FSPCE(X)** **→** **Search persistence (M)** **→** **originality (Y)**		
**Moderator variables**	**Effect**	**SE**	**95% CI**
Low pandemic exposure, low prosocial motivation	0.15	0.11	[−0.01, 0.41]
Low pandemic exposure, average prosocial motivation	0.06	0.06	[−0.03, 0.21]
Low pandemic exposure, high prosocial motivation	−0.03	0.07	[−0.19, 0.12]
Average pandemic exposure, low prosocial motivation	0.24	0.12	[0.02, 0.50]
Average pandemic exposure, average prosocial motivation	0.10	0.08	[−0.05, 0.27]
Average pandemic exposure, high prosocial motivation	−0.04	0.10	[−0.25, 0.16]
High pandemic exposure, low prosocial motivation	0.33	0.17	[0.03, 0.66]
High pandemic exposure, average prosocial motivation	0.14	0.10	[−0.06, 0.35]
High pandemic exposure, high prosocial motivation	−0.06	0.14	[−0.33, 0.21]

*N = 134. CI, confidence interval; FSPCE, family's support of perseverance in creative efforts. Conditional indirect effect are shown with two moderators combined at average value (average level), 1 SD above (high level) and below (low level) the mean. Indirect effects are significant when confidence interval doesn't contain zero*.

## Discussion

Our study makes several contributions to the existing literature on children's creativity. First, originality is important to children. However, as far as we know, only a few studies have been conducted on the influence of search behavior on children's creativity based on the relationship between knowledge assessment or diversified information/knowledge and originality. This study first points out the potential relationship between children's search behavior and the originality of their creative drawings. This finding indicates that when children take part in a task that needs hours to complete, search persistence would promote the originality of those with low prosocial motivation. This finding is in line with a previous study, which found that exposure to diverse information resulted in higher divergent thinking (Clapham, 2001). To the best of our knowledge, the current study is the first to introduce children's searching behaviors into the model, thereby deepening our understanding of children's originality and enabling us to find ways on how to foster children's creativity.

Second, this study also brings new insights into the effects of family climate and pandemic exposure on children's originality. Children's beliefs, motivations, and performance are affected by the amount of support they receive from their parents (Alberto Valdes-Cuervo et al., 2020). In creativity research, family climate, such as encouragement to experience originality and variety (Bloom and Sosniak, 1981; Gardner, 1993; Csikszentmihalyi, 1999; Foster, 2004), encouragement to embrace non-conformism (Miller and Gerard, 1979; Runco and Albert, 1985; Gardner, 1993; Gute et al., 2008), and even encouragement to fantasize (Dacey, 1989; Foster, 2004), have gained research attention in the past years. However, only a few studies have been published on FSPCE, including that of Kwaśniewska et al. (2018). Thus, its influence mechanism on how family climate affects creativity remains unclear. The current study fills this gap and demonstrates that FSPCE has a positively indirect impact on originality through children's search persistence in moderate-high level of pandemic exposure and low-moderate level of prosocial motivation condition. Thus, this study provides a more comprehensive understanding of the creative family climate. It also enriches the previous conclusions that families can either foster or inhibit students' creativity development (Ren et al., 2017) and highlights the importance of a creative family climate during a period dominated by a disaster event.

Some studies have pointed out the positive side of prosocial motivation on creativity (Forgeard and Mecklenburg, 2013). For example, a study found that children with prosocial behavior depicted more creative drawings (rated by detailed drawing elements, such as colors and moving figures) than their less prosocial counterparts (Zee et al., 2020). In contrast, our study contributes to the literature by highlighting the dark side of prosocial motivation on creativity. The main reason might be attributed to the fact that people with high prosocial motivation tend to think from others' perspectives, put greater value on usefulness, and deviate less from routine (Beersma and De Dreu, 2005), which restrict their thinking framework at the expense of originality. This is especially the case in our study, given that the creative drawings depict the ways by which we can protect humans against COVID-19, which is a serious challenge in the real world. Thus, even children with high search persistence and prosocial motivation may be driven to pay attention to the feasibility of generating useful ideas in order to solve such a real-world problem. All these efforts will certainly decrease their level of originality.

Our study also has several implications for children's creativity and family caring. First, the current study revealed the significance of family climate on children's behaviors and originality. Thus, parents need to encourage and support their children when they are exploring their “environment” or trying out new things. For example, parents can tell their children, “Try to change your mind, think of other ways” or “Give it another try, it's ok.” Second, during the COVID-19 pandemic, exposure to the pandemic has caused major changes in the daily lives of children and the social infrastructure they can access and use. Thus, parents should give children more support in terms of reducing the intense emotions related to the event and inspire them to practice greater perseverance while completing a task at hand. Parents are also responsible for finding various ways to influence their children's creativity during a disaster, and one approach is to encourage creative effort.

Third, the results suggested prosocial motivation's inhabitation to originality. For teachers and parents, they should take note of the “dark side” of prosocial motivation. In particular, children with high prosocial motivation may be too realistic and, therefore, fail to break away from the traditional framework, thus sacrificing their originality. Hence, teachers and parents should not deliberately emphasize the usefulness of an idea in the children's daily lives. Especially when the child has an original idea, he/she should be encouraged to simply express it. Furthermore, their efforts should be recognized rather than criticized and measured against the standards of practice.

Our study, however, has several limitations. First, we assessed originality just used one kind of score. It would be more convincing to use multiple tasks to assess originality. However, when we collected data COVID-19 was very serious in China, so collect more comprehensive data is a really challenge for us. Moreover, the content of the drawings of this study is about anti-COVID-19, which makes other dimensions of creativity (flexibility, and fluency) not appropriate for the assessment. In additional, the students' lack of necessary experience of anti-COVID-19 in the real world prevent us regarding usefulness as another dimension of their creativity. Thus, in the future study, more tasks will be needed to assess students' originality and creativity. Second, main variables were assessed by self-reporting. Although the results of Harman's single factor test suggest that common method bias is not of great concern, future research may consider minimizing this potential problem by using experiment design to explore casual relationships between precursors and originality. Third, creativity is domain-specific, but in this study, only creative drawings are analyzed. Whether search persistence can improve the originality of different creative tasks, such as creative dancing, awaits further exploration. Moreover, the topic or theme of the creative drawings used in this study is to protect human beings from the COVID-19 pandemic. This task comes from the real world rather than a fully imaginative drawing task in a lab study. As such, the role of knowledge in the creative process might be different. This suggests that comparative studies in the future might be necessary in order to gain a more comprehensive conclusion. Forth, all children in the study came from China. The traditional Chinese Confucian culture highlights conformity, which may enlarge the negative effect of prosocial motivation on originality. Thus, whether these findings could be generalized to other cultures requires further verification. Moreover, it is important to unravel the cultural influence on originality performance among children. Last but not least, we did not investigate other potential motivations that could direct the creative process, such as the pro-self motivation that values independence and the critical attitudes that are believed to generate more original and better ideas (Beersma and De Dreu, 2005). Thus, future studies can compare the impacts of pro-self and prosocial motivation on originality using the same model.

## Data Availability Statement

The raw data supporting the conclusions of this article will be made available by the authors, without undue reservation.

## Ethics Statement

Ethical review and approval was not required for the study on human participants in accordance with the local legislation and institutional requirements. Written informed consent to participate in this study was provided by the participants' legal guardian/next of kin.

## Author Contributions

BS participated in the study design, data analysis, and manuscript writing. ZX participated in the study design, data collecting, and the manuscript writing. MY participated in the study design and the manuscript writing. CT participated in the study design, data collecting, data analysis, and writing. All authors contributed to the article and approved the submitted version.

## Conflict of Interest

The authors declare that the research was conducted in the absence of any commercial or financial relationships that could be construed as a potential conflict of interest.

## Appendix

Exemplars of drawings.
